# Arms race of temporal partitioning between carnivorous and herbivorous mammals

**DOI:** 10.1038/s41598-018-20098-6

**Published:** 2018-01-29

**Authors:** Yonghua Wu, Haifeng Wang, Haitao Wang, Jiang Feng

**Affiliations:** 1 School of Life Sciences, Northeast Normal University, 5268 Renmin Street, Changchun, 130024 China; 2 Jilin Provincial Key Laboratory of Animal Resource Conservation and Utilization, Northeast Normal University, 2555 Jingyue Street, Changchun, 130117 China; 3 Department of Bioengineering, Stanford University, Stanford, California 94305 USA; 4 Jilin Engineering Laboratory for Avian Ecology and Conservation Genetics, Northeast Normal University, 5268 Renmin Street, Changchun, 130024 China; 5 Jilin Agricultural University, 2888 Xincheng Street, Changchun, 130118 China

## Abstract

Reciprocal coevolutionary changes in predation and anti-predator behaviours have long been hypothesized, but evolutionary-scale evidence is rare. Here, we reconstructed the evolutionary-scale changes in the diel activity patterns of a predator-prey system (carnivorous and herbivorous mammals) based on a molecular phyloecological approach, providing evidence of long-term antagonistic coevolutionary changes in their diel activities. Our molecular reconstruction of diel activity patterns, which is supported by morphological evidence, consistently showed that carnivorous mammals were subjected to a shift from diurnality to nocturnality, while herbivorous mammals experienced a shift from nocturnality to diurnality during their evolutionary histories. A shift in the diel activity of the herbivores as a result of carnivore avoidance is hypothesized based on molecular, morphological and behavioural evidence, and our results suggest an evolutionary-scale arms race of diel activity shifts between carnivorous and herbivorous mammals.

## Introduction

Interactions between carnivorous and herbivorous mammals, representing one of the classic coevolutionary systems, lead to long-term reciprocal evolutionary changes in predation and anti-predator behaviours^1^. Among carnivorous mammals, felids (Felidae) and canids (Canidae) are the main predators of herbivorous mammals (e.g., ungulates)^2^. These carnivores (felids and canids) and ungulates show differentiated diel activity patterns, with most felids and canids being mainly nocturnal, while ungulates are primarily diurnal^3,4^. Given the differentiation of their diel activity patterns, one possibility is that the diurnality of ungulates may have evolved as an anti-predator behaviour. Previous behavioural ecological studies have shown that the diel activity patterns of prey are strongly influenced by their predators, and prey species are capable of adjusting their activity times to avoid predators, as observed in insects, fish, birds and mammals^5–7^. For instance, Norway rats (*Rattus norvegicus*) were found to shift from nocturnality to diurnality to avoid predation by nocturnal red foxes (*Vulpes vulpes*)^8^. Furthermore, ungulates (e.g., buffalo, kudu and giraffe) in African savannah were shown to be capable of avoiding the hours of the day with a high predation risk from lions, suggesting that predation pressure was the key to the switch in their activity patterns^9^. In particular, ungulates such as buffalo and kudu that are more active at night in the absence of predators become more active in the day time after the reintroduction of large nocturnal predators (lions and hyaenas)^10^. In addition to the influence of predators on the diel activity changes of their prey, prey species also have an effect on the diel activities of their predators^11^. The known reciprocal influences of predators and prey on the changes in the diel activity patterns of these animals may imply that, at an evolutionary scale, there could be reciprocal coevolutionary changes in diel activity patterns between carnivorous mammals and ungulates (referred to as the antagonistic coevolution hypothesis hereafter). However, such relationships are difficult, or sometimes impossible to test because behaviours are less likely to be preserved in the fossil record than other characteristics.

**Figure 1:**
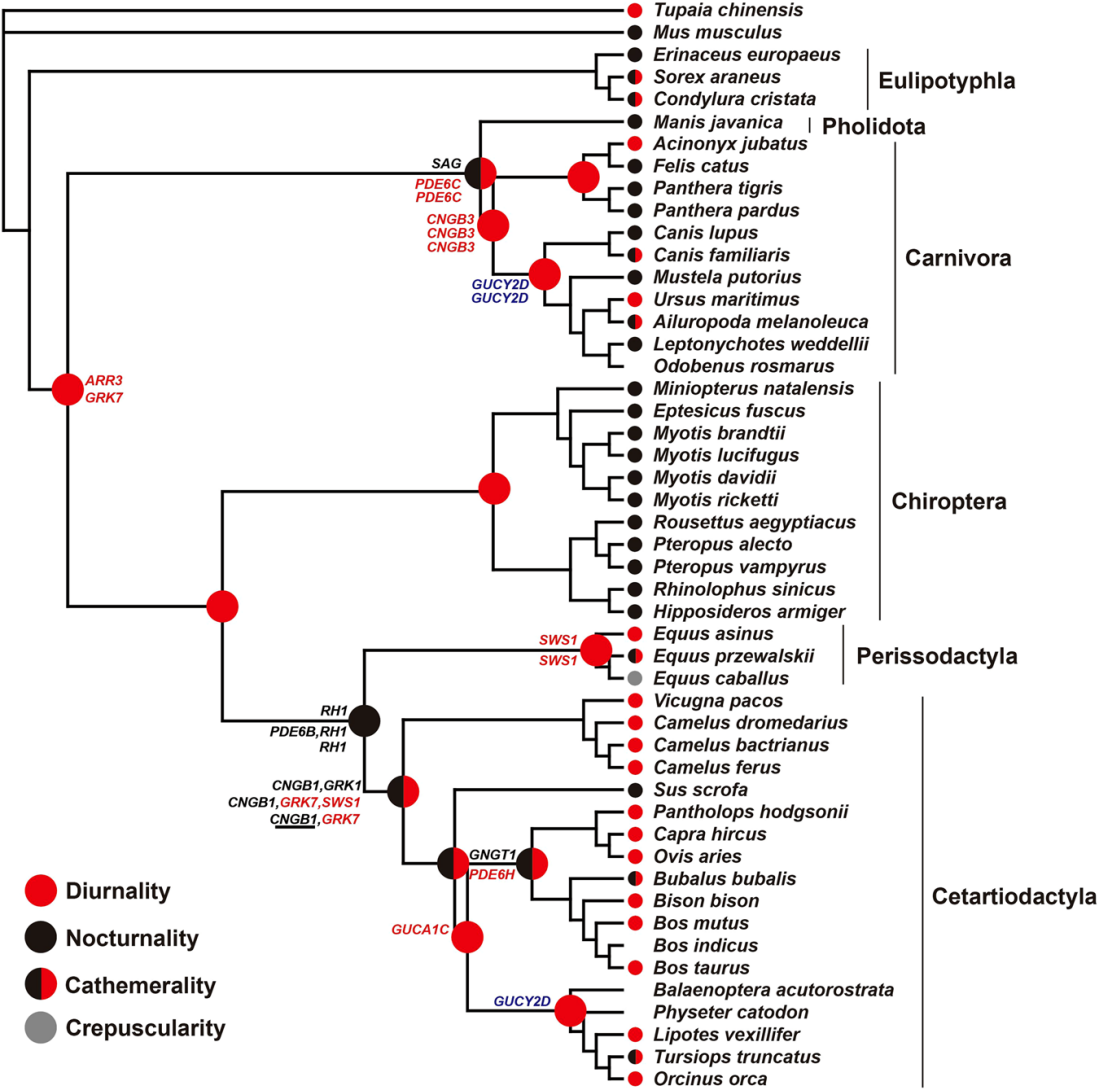
Reconstruction of the diel activity patterns. Colored dots show the reconstructed diel activity patterns of ancestral nodes. The available diel activity patterns of living species are also shown. The positively selected bright-light vision genes (red), the positively selected dim-light vision genes (black) and both suggest diurnality, nocturnality and cathemerality, respectively. Lack of positive selection signals along certain branches is treated as the retention of the diel activity patterns of their most recent common ancestors. The positively selected photoresponse recovery gene, *GUCY2D*, is involved in both dim-light vision and bright-light vision and is shown in blue. The positive selection genes found along certain branches based on PAML, BUSTED and BS-REL are respectively shown from top to bottom. The diel activity patterns of living species are based on published literature^3,51,52^ or Animal Diversity Web (http://animaldiversity.org/). Only species mainly used in this study are shown and their phylogenetic relationships follow previous studies^43–49^. Underline shows positive selection signal is lost when phylogenetic uncertainty is taken into account.

**Figure 2:**
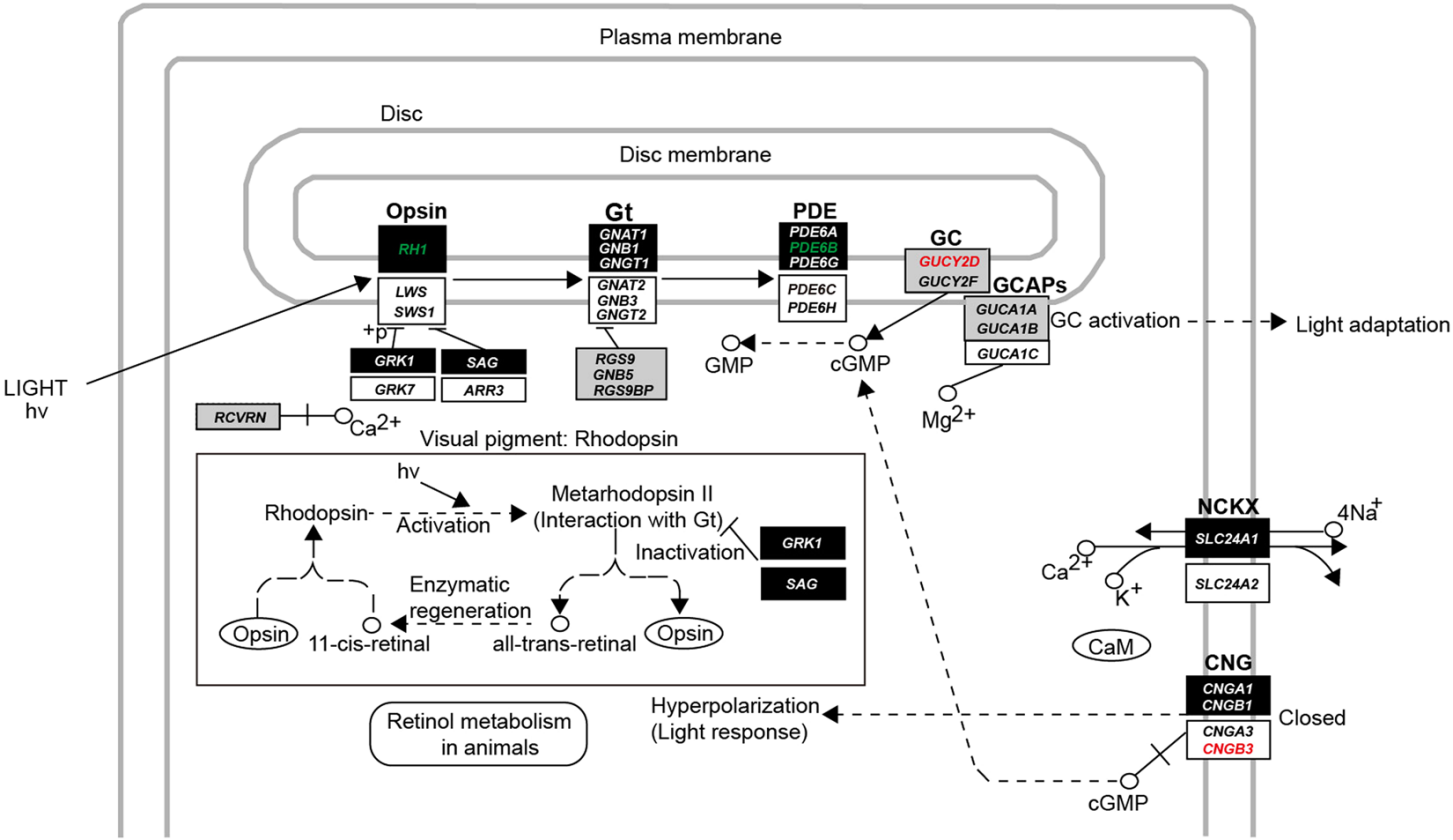
Positively selected genes involved in the phototransduction pathway in rods (according to KEGG pathway: map04744). For convenience, the genes involved in the phototransduction pathway in cones are also shown. Dark rectangles, white rectangles and grey rectangles show genes involved in the phototransduction pathway of rods, cones and both, respectively^18,19^. Only the positively selected genes identified in carnivores (red) and ancestral Euungulata (green) are shown. Solid line shows direct interaction and dashed line shows indirect interaction.

Recently, a molecular phyloecological approach has been developed to reconstruct the ancestral trait status of diel activity patterns in mammals and birds. This method is sensitive in discriminating different diel activity patterns using genes involved in the cone/rod phototransduction pathway: diurnality is characterized by enhanced selections for bright-light vision genes (cone-expressed genes) and nocturnality is characterized by enhanced selections for dim-light vision genes (rod-expressed genes)^12–14^. To test the antagonistic coevolution hypothesis of the evolution of diel activity between carnivores and ungulates, in the present study, we used this molecular phyloecological approach and employed both restricted models (PAML)^15^ and unrestricted models (BUSTED, BS-REL)^16,17^ to identify intensified selection of bright-light vision genes and dim-light vision genes involved in the cone/rod phototransduction pathway^18–20^ along various branches of carnivorous mammals and ungulates, in the context of the Laurasiatheria phylogeny (Figs 1 and 2, Supplementary Table S1). This approach enabled us to track the long-term evolutionary changes in the diel activity patterns of carnivorous and herbivorous mammals for the first time. Our results consistently demonstrated opposite shifts in diel activity between ungulates and carnivores, and a shift in the diel activity of ungulates as a result of carnivore avoidance was inferred based on multiple lines of evidence. Our study supports the long-term antagonistic coevolution of temporal partitioning between carnivorous and herbivorous mammals.

## Results and Discussion

### Carnivores: shifting from diurnality to nocturnality

**Figure 3:**
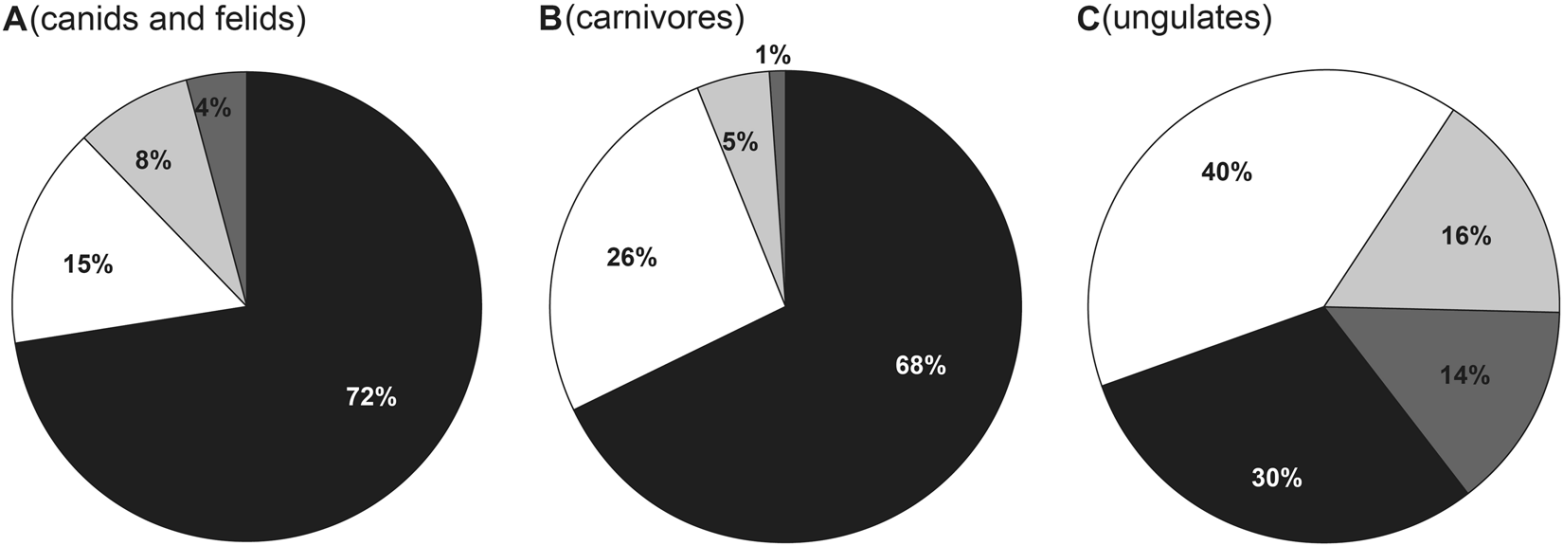
Estimated proportions of species with different diel activity patterns in canids and felids (**A**), carnivorous mammals (**B**) and ungulates (odd-toed ungulates and even-toed ungulates combined) (**C**). Nocturnal (black), diurnal (white), cathemeral (light grey) and crepuscular (dark grey) are shown in different colors. Totally, 488 terrestrial species data are used (including 243 out of 281 carnivoran species and 245 out of 257 ungulate species according to Wilson & Mittermeier (2009)^2^). The diel activity pattern data are based on one published study^3^.

Most extant carnivorous mammals are typically nocturnal (68%) (Fig. 3, Supplementary Fig. S1, Supplementary Tables S2 and S3), while the diel activity patterns of ancestral carnivorous mammals remain unknown. To determine the diel activity patterns of ancestral carnivores, we analysed the adaptive evolution of 33 phototransduction genes along the ancestral carnivore branch using PAML, BUSTED and BSREAL. PAML, BUSTED and BSREAL consistently showed only one gene, *CNGB3*, to be under positive selection, which was independent of phylogenetic uncertainty and variations in the initial values of kappa and ω (Figs 1 and 2, Table 1, Supplementary Tables S4 and S5). *CNGB3* is a bright-light vision gene that is involved in the activation of the cone phototransduction pathway. The only identified positively selected bright-light vision gene suggests enhanced visual acuity under bright-light conditions and, hence, strongly indicates predominate diurnality of ancestral carnivores. Given the predominate diurnality of ancestral carnivores, the prevalence of nocturnality in extant carnivores is a derived trait. To determine when the shift from diurnality to nocturnality occurred within carnivorous mammals, we further analysed positive selection along the branches leading to Feliformia and Caniformia, and no positively selected genes (PSGs) were detected (Fig. 1). This result suggests that the shift from diurnality to nocturnality may have occurred in other subgroups within carnivorous mammals. Indeed, among Felidae and Canidae, which are the main predators of ungulates, approximately 75% and 69% of extant species are nocturnal (Supplementary Tables S3), respectively, suggesting that a transition to nocturnality occurred within these groups. Future studies incorporating more taxa of felids and canids may help to identify the taxa that underwent the transition to nocturnality.

**Table 1:** Positively selected genes identified based on the branch-site model of PAML.

Taxa/Genes	Parameter estimates	2∆L	df	*P*-value	Positively selected sites
**Scrotifera**					
*ARR3*	*p*_0_ = 0.746 *p*_1_ = 0.217 *p*_*2a*_ = 0.029 *p*_*2b*_ = 0.008	6.23	1	0.013	12 H, 37 T, 158 I,
	*ω*_0_ = 0.138 *ω*_1_ = 1.000 ***ω***_***2a***_ = **57.886** ***ω***_***2b***_ = **57.886**				166 K, **192 G**
**Ancestral Euungulata**					
*RH1*	*p*_0_ = 0.938 *p*_1_ = 0.054 *p*_*2a*_ = 0.007 *p*_*2b*_ = 0.000	3.98	1	0.046	**200 M**, 269 T
	*ω*_0_ = 0.029 *ω*_1_ = 1.000 ***ω***_***2a***_ = **999.000** ***ω***_***2b***_ = **999.000**				
**Cetartiodactyla**					
*CNGB1*	*p*_0_ = 0.904 *p*_1_ = 0.089 *p*_*2a*_ = 0.007 *p*_*2b*_ = 0.001	15.97	1	6.43E-05	46 H, 63 E, **263 R**
	*ω*_0_ = 0.051 *ω*_1_ = 1.000 ***ω***_***2a***_ = **999.000** ***ω***_***2b***_ = **999.000**				343 Q, 345 I, 389 L
*GRK1*	*p*_0_ = 0.898 *p*_1_ = 0.095 *p*_*2a *_= 0.007 *p*_*2b*_ = 0.001	16.76	1	4.25E-05	103 L, 160 Q, 183 K
	*ω*_0_ = 0.051 *ω*_1_ = 1.000 ***ω***_***2a***_ = **998.999** ***ω***_***2b***_ = **998.999**				295 R, **392 S**, 414 E
					438 R, 495 S
**Common ancestors (Bovidae + Cetacea)**					
*GUCA1C*	*p*_0_ = 0.891 *p*_1_ = 0.081 *p*_*2a*_ = 0.026 *p*_*2b*_ = 0.002	6.5	1	0.011	**13 L**
	*ω*_0_ = 0.298 *ω*_1_ = 1.000 ***ω***_***2a***_ = **74.669** ***ω***_***2b***_ = **74.669**				
**Cetacea**					
*GUCY2D*	*p*_0_ = 0.941 *p*_1_ = 0.056 *p*_*2a*_ = 0.003 *p*_*2b*_ = 0.000	4.96	1	0.026	**23 G**, 58 I, 62 H, 65 G
	*ω*_0_ = 0.047 *ω*_1_ = 1.000 ***ω***_***2a***_ = **42.656** ***ω***_***2b***_ = **42.656**				
**Bovidae**					
*GNGT1*	*p*_0_ = 0.986 *p*_1_ = 0.000 *p*_*2a*_ = 0.014 *p*_*2b*_ = 0.000	5.9	1	0.015	**40 V**
	*ω*_0_ = 0.059 *ω*_1_ = 1.000 ***ω***_***2a***_ = **999.000** ***ω***_***2b***_ = **999.000**				
**Perissodactyla**					
*SWS1*	*p*_0_ = 0.830 *p*_1_ = 0.131 *p*_*2a*_ = 0.034 *p*_*2b*_ = 0.005	6.23	1	0.013	17 M, **29 A**, **70 A**
	*ω*_0_ = 0.129 *ω*_1_ = 1.000 ***ω***_***2a***_ = **8.737** ***ω***_***2b***_ = **8.737**				
**Carnivora**					
*CNGB3*	*p*_0_ = 0.853 *p*_1_ = 0.137 *p*_*2a*_ = 0.009 *p*_*2b*_ = 0.001	7.17	1	0.007	**107 E**, 213 V
	*ω*_0_ = 0.123 *ω*_1_ = 1.000 ***ω***_***2a***_ = **999.000** ***ω***_***2b***_ = **999.000**				
**Common ancestors (Carnivora + Pholidota)**					
*SAG*	*p*_*0*_ = 0.881 *p*_*1*_ = 0.108 *p*_*2a*_ = 0.009 *p*_*2b*_ = 0.001	4.19	1	0.041	59 Q, **224 T**
	*ω*_*0*_ = 0.048 *ω*_*1*_ = 1.000 ***ω***_***2a***_ = **30.806** ***ω***_***2b***_ = **30.806**				

For convenience, only the ω values of foreground branches are shown. The positively selected sites with a high posterior probability support (>0.900) are shown in bold.

2∆L: twice difference of likelihood values between the modified model A and the corresponding null model with the ω = 1 fixed in the foreground branches; df: degrees of freedom; proportion of sites and their corresponding ω values in four site classes (p_0_, p_1_, p_2a_ and p_2b_) of the branch-site model are shown.

To further determine whether the predominate diurnality of ancestral carnivores was derived or retained from their progenitors, we subsequently analysed positive selection in the branch of the common ancestor of Carnivora and Pholidota, the ancestral branch of Scrotifera, which includes four closely related groups (Pholidota, Carnivora, Chiroptera and Euungulata (Perissodactyla and Cetartiodactyla)), and the ancestral branch of Laurasiatheria. For the branch of the common ancestor of Carnivora and Pholidota, one dim-light vision gene (*SAG*) and one bright-light vision gene (*PDE6C*) were found to be under positive selection (Fig. 1, Table 1, Supplementary Tables S4 and S5), suggesting the possibility of cathemerality in this ancestor. In the ancestral Scrotifera branch, two positively selected bright-light vision genes (*ARR3* and *GRK7*) were detected (Fig. 1, Table 1, Supplementary Tables S4), suggesting diurnality. No PSGs were found along ancestral Laurasiatheria branch. The identification of possible cathemerality in the common ancestor of Carnivora and Pholidota suggests that the diurnality of ancestral carnivores is more likely a derived trait.

### Ungulates: shifting from nocturnality to diurnality

Extant ungulates are primarily diurnal^3,4^ (Fig. 3, Supplementary Fig. S1, Supplementary Tables S2 and S3), while the diel activity pattern of ancestral ungulates is unknown. To determine possible evolutionary-scale changes in the diel activity patterns of ungulates, positive selection on phototransduction genes along various branches within ungulates was analysed (Fig. 1). For the ancestral ungulate branch, only two dim-light vision genes (*RH1* and *PDE6B*) were identified as being under positive selection (Fig. 1, Table 1, Supplementary Tables S4 and S5). These two genes are known to be involved in the activation of the rod phototransduction pathway, and their positive selection suggests enhanced sensitivity to the detection of dim light, thus strongly suggesting the nocturnality of ancestral ungulates. In light of this finding, our subsequent identification of positively selected bright-light vision genes along various branches within Perissodactyla and Cetartiodactyla (Fig. 1, Table 1, Supplementary Tables S4 and S5) suggests the occurrence of a gradual transition from nocturnality in ancestral ungulates to diurnality in derived groups. To further evaluate whether the nocturnality of ancestral ungulates is a derived characteristic or was retained from their progenitors, analyses of positive selection were conducted along the branch of the common ancestor of ungulates and their sister taxon, bats, and no PSGs were found. This result could suggest that the common ancestor of ungulates and bats may have retained the diel activity pattern of its progenitor, the ancestor of Scrotifera, which was determined to be diurnal (Fig. 1). Given the possible diurnality of the common ancestor of ungulates and bats, the nocturnality of ancestral ungulates is more likely a derived trait.

### The diel activity shift of ungulates as a possible carnivore avoidance strategy

Given the opposite shifts in diel activity patterns observed between ungulates (shifting from nocturnality to diurnality) and their predators, the carnivores (e.g., felids and canids, shifting from diurnality to nocturnality), we hypothesize that the diel activity shift of ungulates may have evolved as a carnivore avoidance strategy, considering that previous behavioural ecological observations have demonstrated that prey (e.g., ungulates) frequently adjust their diel activities to avoid their predators^5–10^. If this is the case, we would expect that a taxon that is free from predation by carnivores would not show a similar change in the diel activity to that found in ungulates. To test this expectation, we reconstructed the evolution of the diel activity of bats, a sister taxon of ungulates that is not subject to predation by carnivores. Our analysis of positive selection along the ancestral bat branch showed no PSGs, suggesting that ancestral bats may have retained the visual adaption of the common ancestor of bats and ungulates, which was reconstructed as diurnal, as described above (Fig. 1). Subsequent analysis of selection intensity for all 33 phototransduction genes along the ancestral bat branch using RELAX showed one bright-light vision gene (*CNGB3*) and one photoresponse recovery gene (*RCVRN*) to be under intensified selection (*k* > 1) relative to the ancestral ungulate branch (Supplementary Table S6). These findings may suggest that ancestral bats exhibited relatively enhanced bright-light vision, with an increased motion detection ability, providing additional evidence of their diurnality. Given the reconstructed diurnality of ancestral bats, there should have been a shift from diurnality to nocturnality during bat evolution, since almost all extant bats are nocturnal, possibly due to predation by diurnal raptors^21^. Thus, our results suggested that bats do not show a similar change in diel activity to that found in ungulates, providing evidence supporting our hypothesis of the evolution of the diel activity shift of ungulates as a carnivore avoidance behaviour.

The timing of the origin and evolution of carnivorous mammals and ungulates is compatible with our hypothesized shift in the diel activity of ungulates as a carnivore avoidance strategy. Palaeobiological evidence shows that carnivorous mammals and ungulates have coexisted since the Palaeocene^22,23^. The earliest carnivorous mammals (including Viverravidae and Miacidae) have been identified from the middle Palaeocene to the late Palaeocene^24^, and diverse carnivoraformes taxa evolved and radiated in the Eocene, with the crown Carnivora (e.g., Canidae and Felidae) appearing relatively recently, during late Eocene times^25–28^. The late Eocene origins of Canidae and Felidae, which are the main extant predators of ungulates^2^ and exhibit a high proportion of nocturnal species (72%, Fig. 3), may suggest that their transition to nocturnality appeared as early as the late Eocene. Among ungulates, the most primitive examples (Condylarthra) are known from the Palaeocene^22,23^, and the earliest fossil records of Artiodactyla and Perissodactyla appeared in the early Eocene^22,25,29^. The accurate timing of the transitions of the diurnality of extant diurnal ungulates is less well established. Nevertheless, considering that three ungulate groups (Suidae, Cervidae and Bovidae) that are among the principle prey of canids and felids and harbour most diurnal species of extant ungulates (Supplementary Fig. S1) arose in relatively recent times compared with their main predators (Canidae and Felidae), from the early Oligocene to the Miocene^25^, it is possible that their diurnality transition may have occurred as early as the early Oligocene. The relatively late transitions to diurnality in these ungulates compared with the relatively earlier potential transition to nocturnality in their main predators may reflect an evolutionary lag. It should be noted that there are numerous extinct carnivorous mammals^28^ whose diel activity patterns are unknown, and the potential influence of these extinct carnivores on the changes in the diel activity of ungulates is therefore also not known. Given the long-term coexistence of these groups and the possible influence of carnivorous mammals on the changes in the diel activity of ungulates, one possible scenario is that the predation pressures from diurnal ancestral carnivores may have initially forced ancestral ungulates to adopt nocturnality, and a subsequent shift of carnivorous mammals to nocturnality then led to the derived taxa of ungulates to switch back diurnality to avoid their carnivorous predators.

Given the shift in their diel activity during their evolution, the predominate nocturnality of extant carnivores (e.g., Canidae and Felidae) and the primary diurnality of extant ungulates are probably a result of their reciprocal antagonistic coevolution. The reciprocal antagonistic coevolution of diel activity between carnivores and ungulates may have led to their adaptive divergence in terms of relevant vision gene functions. To test this hypothesis, we analysed the relative changes in selection intensity on phototransduction genes in both carnivorous mammals and ungulates (Supplementary Tables S7 and S8). Our results showed that relative to the entire Euungulata clade, the Carnivora clade mainly exhibited intensified selection (*k* > 1) on dim-light vision genes and somewhat relaxed selection (*k < *1) on bright-light vision genes (Supplementary Table S7), whereas the Euungulata clade mainly exhibited relaxed selection on dim-light vision genes and somewhat intensified selection on bright-light vision genes (Supplementary Table S8), suggesting relatively enhanced dim-light vision in carnivores and relatively enhanced bright-light vision in ungulates, which is consistent with behavioural observations showing that modern carnivorous mammals and ungulates are mainly nocturnal and diurnal, respectively.

### Morphological evidence for the reconstructed diel activity patterns

Morphological evidence provides indirect support for our molecular reconstruction of diel activity patterns. In the eye, the tapetum is a specialized tissue adapted to increase retinal sensitivity in dim-light conditions and has been found to exhibit diverse structures and compositions in mammals^30,31.^ Among our three focal taxa, three different types of tapeta are found in the Carnivora (tapetum cellulosum), Euungulata (tapetum fibrosum) and Chiroptera (retinal tapetum)^30,31^. The different tapeta observed in the three groups may suggest multiple independent origins of tapeta in these groups and may imply a lack of tapetum in their common ancestor, consistent with its diurnality inferred based on our molecular results (Fig. 1). Specifically, within Carnivora, although a tapetum cellulosum is present in almost all studied species, it has been found to exhibit different compositions in the two suborders of Carnivora, Feliformia and Caniformia, with riboflavin being observed in the former and zinc cysteine in the latter^30^, suggesting independent evolution of the tapeta in these two groups. Otherwise, if nocturnality and the tapetum are assumed to have existed in ancestral carnivores, the tapeta of its two derived subgroups, Feliformia and Caniformia, would be expected to be the same. However, the different types of tapeta present in these subgroups are inconsistent with this assumption, which may suggest that the ancestral carnivores had not evolved a tapetum, which would be consistent with their inferred diurnality (Fig. 1). The diurnality of the ancestral carnivores is also morphologically supported by a lack of an ossified tympanic bulla in Miacids, which are regarded as the most primitive representatives of Carnivora^25,32^. The lack of an ossified tympanic bulla in Miacids may suggest that their auditory systems are less developed compared with later carnivorans, from which the ossified tympanic bulla appears to have evolved independently in Feliformia and Caniformia^32^. The relatively less developed auditory system of Miacids would be incompatible with a nocturnal lifestyle, which is frequently characterized by enhanced hearing^29,33^.

Unlike the situation in Carnivora, in Euungulata, only one type of tapetum, a tapetum fibrosum composed of collagen, has been identified in both Perissodactyla and Cetartiodactyla^31^, strongly indicating a common origin of the tapetum in these two groups. If this is the case, it may necessarily suggest that the ancestral Euungulata was more likely to have had a tapetum fibrosum, consistent with its inferred nocturnality (Fig. 1).

Bats usually lack a tapetum, with the exception of Old World fruit bats (Pteropodidae)^31,34^, which are known to depend mainly on vision and olfaction for orientation. In pteropodids, a tapetum is present, but it is classified as a retinal tapetum, differing from the choroidal tapeta used by Carnivora and Euungulata^30,31,34^. Considering that pteropodids are phylogenetically nested among echolocating bats^35^, which are not known to have a tapetum^31,34,36^, the tapetum of pteropodids is more likely to have evolved secondarily. Otherwise, if an origin of the retinal tapetum in ancestral bats were to be assumed, most extant bats would be expected to have retained the retinal tapetum, rather than losing it, since almost all of these species are nocturnal and show strong visual adaption (rod-dominated retinas) to dim-light^37–39^. Therefore, ancestral bats may have lacked a tapetum, which is consistent with their inferred diurnality. In addition, the earliest known bat fossil (*Onychonycteris*), from the early Eocene, is believed to lack echolocation ability and is thought to have likely detected its prey using vision^40^, which is normally a characteristic of diurnal taxa, supporting the diurnality of this bat. Our molecular results showed that ancestral bats may have had a relatively enhanced capability for motion detection under bright-light conditions (Supplementary Table S6), which may have facilitated their aerial capture of flying insects during day time.

## Conclusion

Our molecular and morphological data provide a consistent reconstruction of the diel activity patterns of our focal taxa. Accordingly, a shift from diurnality to nocturnality in carnivorous mammals (e.g., felids and canids) and a shift from nocturnality to diurnality in ungulates are identified. The shifts in their diel activity patterns as a result of reciprocal antagonistic coevolution are hypothesized based on multiple lines of evidence. Although we hypothesize that the diurnality of extant ungulates may have evolved as a result of a carnivore avoidance strategy, the possibility of the other various diel activities of ungulates (e.g., nocturnality and cathemerality) resulting from the predation of diurnal and/or cathemeral carnivores remains to be explored. It is more likely that the diel activity patterns of individual species may evolve as a trade-off under the effects of specific predation pressures or other possible factors (e.g., competition). Moreover, our study includes partial representative taxa of our focal taxa. Future studies including more taxa and incorporating information about different factors may lead to a more robust reconstruction of diel activities that helps to elucidate the specific evolution of the diel activity patterns of different taxa.

## Materials and Methods

### Taxa and sequences covered

In this study, all species of carnivorous mammals (Carnivora), herbivorous mammals (Perissodactyla and Cetartiodactyla) and closely related taxa (Pholidota and Chiroptera) were incorporated depending on sequence availability. Species from groups of distant relatives, such as Eulipotyphla and/or Euarchontoglires, were included as outgroups. For all of our focal taxa, the coding sequences of 33 phototransduction genes, involved in both the rod and cone phototransduction pathways, were downloaded from GenBank (Supplementary Table S1). For each gene, the longest transcript variant was used when multiple transcript variants were available. We aligned the gene sequences using the online software webPRANK^41^ (http://www.ebi.ac.uk/goldman-srv/webprank/), which is believed to reduce false-positive results of positive selection analyses by generating a more reliable alignment than other software^42^. The sequence alignments were inspected manually for quality, and individual sequences with low identities, long indels, multiple ambiguous bases Ns, and/or too short a length were removed or replaced by other relevant transcript variants. After this pruning, high-quality alignments were constructed, and their translated protein sequences were confirmed through Blast searches against the non-redundant protein sequence (nr) database.

### Analyses of positive selection

We used various models implemented in different software (PAML, BUSTED and BS-REAL) for positive selection analyses. These models incorporate different assumptions and present various degrees of power for detecting positive selection. The uses of these different models would help to examine the robustness of our results. For our analyses, an unrooted species tree (Fig. 1) was constructed based on published studies^43–49^. The species included for each gene were subject to change depending on sequence availability (Please see Supplementary Table S1 for details).

In PAML, we used a branch model and a branch-site model, which were implemented in the Codeml program^15^, for our positive selection analyses. These models use a codon-based maximum likelihood method to estimate the ratio of non-synonymous to synonymous substitutions per site (dN/dS or ω), and ω < 1, ω = 1 and ω > 1 suggest purifying selection, neutral evolution and positive selection, respectively. Using these two models, positive selection was analysed along various branches of interest. In these analyses, each of our focal branches was used as the foreground branch, while all others were treated as background branches, and likelihood ratio tests (LRT) were then applied to determine statistical significance by comparing the null models with the corresponding alternative models.

Branch model. We employed a two-rate branch model to identify positively selected branches of interest. The two-rate branch model allows ω to vary between foreground branches and background branches, and its goodness of fit was determined using the LRT, based on comparison with a one-rate model that assumes a single unchanged ω value for all branches. If the LRT was determined to be statistically significant, the two-ratio model was then compared with the two-ratio model with a constraint of ω = 1 under the LRT to further determine whether the ω value of our focal foreground branch was greater than 1 with statistical support.

Branch-site model. We used a branch-site model (Test 2) to detect positively selected sites along our focal branches. Test 2 compares a modified model A with its corresponding null model with ω = 1 constrained. In the modified model A, four classes of sites are assumed, with site class 0 (0 < ω_0_ < 1) and site class 1 (ω_1_ = 1) representing evolutionarily conserved and evolutionarily neutral codons, respectively, along both background branches and foreground branches, while site classes 2a and 2b represent evolutionarily conserved (0 < ω_0_ < 1) or neutral (ω_1_ = 1) codons, respectively, along background branches but are allowed to be under positive selection (ω_2_ > 1) along foreground branches. Positively selected sites were identified via an implemented Bayes Empirical Bayes method.

In addition to PAML, we employed the branch site-random effects likelihood (BS-REL) test^16^ and the branch site-unrestricted statistical test for episodic diversification (BUSTED)^17^ to analyse positive selection for our focal branches. These two methods (BS-REL and BUSTED) mainly differ from PAML in their different model assumptions used. In PAML, all branches are grouped *a priori* into foreground and background branches, and only the foreground branches are allowed to be under positive selection, while the background branches are constrained to being negatively selected or neutral (restricted model). Unlike PAML, BS-REL does not require partitioning of foreground and background branches and allows the occurrence of positive selection across the tree (unrestricted model). Upon analysis, three ω categories (ω1, ω2 and ω3) are assumed, representing strongly and weakly conserved and positively selected sites of each branch, respectively, and the ω values of the three ω categories with the corresponding site proportions are calculated. Positively selected branches were identified based on a sequential likelihood ratio test.

BUSTED is mainly distinguished from BS-REL by its ability to test positive selection on particular lineages (interested) without restriction of the occurrence of positive selection in the rest of the tree^17^. BUSTED normally requires *a prior* partitioning of branches into foreground branches and background branches and is considered to show increased power to identify positive selection compared with BS-REL. In these analyses, the foreground branches are allowed to undergo positive selection (alternative model), and a likelihood ratio statistic is then used to determine fitness based on comparison with the null model, which does not allow positive selection of the foreground branches, with a constraint of ω3 = 1. Bonferroni multiple testing correction was used to adjust *P* values.

### Robustness tests of positively selected genes

Positively selected genes identified by PAML were further examined for robustness by taking phylogenetic uncertainty and the variations of the initial values of kappa and omega into account. Compared with the species tree that we initially used (Fig. 1), there was uncertainty in the phylogenetic positions of some taxa, regarding the phylogenies of Carnivora and Cetartiodactyla from *10 kTrees* data (http://10ktrees.fas.harvard.edu/). Specifically, compared with the tree that we initially used, the 10 *kTrees* data showed an exchange of phylogenetic positions between the genus *Sus* and one clade including the genera *Camelus* and *Vicugna* in Cetartiodactyla, in addition to an exchange of the phylogenetic positions of Ursidae and Mustelidae within Carnivora. Given the new phylogenetic relationships, the positive selection signals of those PSGs were analysed. In addition, to examine the dependency of the detected PSGs on the variations in the initial values of kappa and omega, we used two different initial values of kappa (kappa = 0.5, 3.0) and two different initial values of omega (omega = 0.5, 2.0) for our positive selection analyses. Similar to the PAML analysis, in the BS-REL and BUSTED analyses, the new phylogeny was also used to examine the effects of the phylogenetic uncertainties on our results. In addition, we ran BS-REL and BUSTED a second time to confirm the identified positive selection signals.

### Analyses of selection intensity

The changes in the relative selection intensity on phototransduction genes were analysed by using RELAX^50^, which is available from the Datamonkey webserver (http://test.datamonkey.org/relax). Given *a priori* partitioning of test branches and reference branches in a codon-based phylogenetic framework, RELAX is used to calculate a selection intensity parameter value (*k*) and its statistical significance, with *k* > 1 showing intensified selection and *k* < 1 showing relaxed selection. Intensified selection is expected to show ω categories away from neutrality (ω = 1), while relaxed selection is expected to show ω categories converging to neutrality (ω = 1). LRT is used to determine statistical significance by comparing an alternative model to a null model. The null model assumes *k* = 1 and the same ω distribution of both test and reference branches, while the alternative model assumes that *k* is a free parameter, and the test and reference branches exhibit different ω distributions.

## Electronic supplementary material

Supplementary file

Dataset 1

Dataset 2
