# Supplementary material for: Arms race of temporal partitioning between carnivorous and herbivorous mammals
Source: Sci Rep. 2018 Jan 29;8:1713. doi: 10.1038/s41598-018-20098-6 (PMC5789060; doi:10.1038/s41598-018-20098-6)
Supplement: Supplementary file 1 — Supplementary file [file 41598_2018_20098_MOESM1_ESM.doc]

**Arms race of temporal partitioning between carnivorous and herbivorous mammals**

### Yonghua Wu, Haifeng Wang, Haitao Wang, Jiang Feng

**
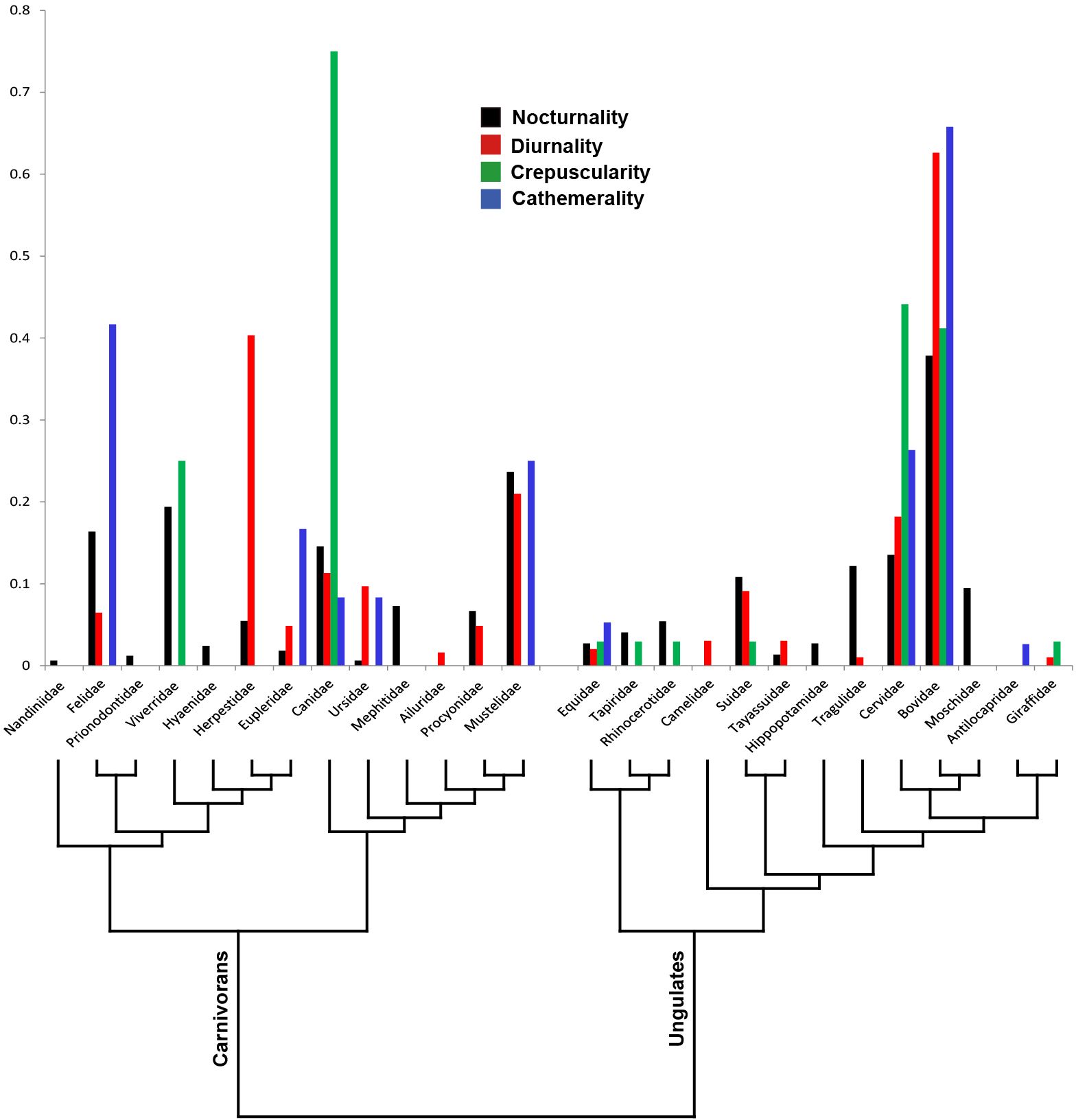
**

**Supplementary Figure S1 The proportions of species with different diel activity patterns**. The proportions of species with different diel activity patterns are calculated by dividing the number of species with one certain diel activity pattern within one certain family by the total number of species with the same diel activity pattern within each of two groups (carnivorans and ungulates). The proportions are calculated for carnivorans and ungulates separately. The diel activity data are based on one published study ^3^ (please also see Supplementary Table S2). The phylogenetic relationships of families followed one published study ^47^.

**Supplementary Table S3 The proportions of species with different diel activity patterns within each family.** The diel activity data are based on one published study ^3^.

| **Family** | **Nocturnal** | **Diurnal** | **Crepuscular** | **Cathemeral** |
| --- | --- | --- | --- | --- |
| Nandiniidae | 1.00 | 0.00 | 0.00 | 0.00 |
| Felidae | 0.75 | 0.11 | 0.00 | 0.14 |
| Prionodontidae | 1.00 | 0.00 | 0.00 | 0.00 |
| Viverridae | 0.97 | 0.00 | 0.03 | 0.00 |
| Hyaenidae | 1.00 | 0.00 | 0.00 | 0.00 |
| Herpestidae | 0.26 | 0.74 | 0.00 | 0.00 |
| Eupleridae | 0.38 | 0.38 | 0.00 | 0.25 |
| Canidae | 0.69 | 0.20 | 0.09 | 0.03 |
| Ursidae | 0.13 | 0.75 | 0.00 | 0.13 |
| Mephitidae | 1.00 | 0.00 | 0.00 | 0.00 |
| Ailuridae | 0.00 | 1.00 | 0.00 | 0.00 |
| Procyonidae | 0.79 | 0.21 | 0.00 | 0.00 |
| Mustelidae | 0.71 | 0.24 | 0.00 | 0.05 |
| Equidae | 0.29 | 0.29 | 0.14 | 0.29 |
| Tapiridae | 0.75 | 0.00 | 0.25 | 0.00 |
| Rhinocerotidae | 0.80 | 0.00 | 0.20 | 0.00 |
| Camelidae | 0.00 | 1.00 | 0.00 | 0.00 |
| Suidae | 0.44 | 0.50 | 0.06 | 0.00 |
| Tayassuidae | 0.25 | 0.75 | 0.00 | 0.00 |
| Hippopotamidae | 1.00 | 0.00 | 0.00 | 0.00 |
| Tragulidae | 0.90 | 0.10 | 0.00 | 0.00 |
| Cervidae | 0.19 | 0.34 | 0.28 | 0.19 |
| Bovidae | 0.22 | 0.48 | 0.11 | 0.19 |
| Moschidae | 1.00 | 0.00 | 0.00 | 0.00 |
| Antilocapridae | 0.00 | 0.00 | 0.00 | 1.00 |
| Giraffidae | 0.00 | 0.50 | 0.50 | 0.00 |

| **Supplementary Table S4 Positively selected genes identified by BUSTED.** For analyses, each taxon was respectively treated as foreground branch (FG) and others were used as background branches (BG). Only branches of interest with positively selected genes found are shown. | | | | | | | | | | | |
| --- | --- | --- | --- | --- | --- | --- | --- | --- | --- | --- | --- |
| **Taxa/Gene** | **Model** | ***log* L** | **#par.** | **Branch** | **ω1** | **ω2** | **ω3** | ***P*-value** | **Corrected** | |  |
|  |  |  |  | **set** |  |  |  |  | ***P*-value** | |  |
|  |  |  |  |  |  |  |  |  |  | |  |
| **Scrotifera** |  |  |  |  |  |  |  |  |  | |  |
| ***GRK7*** | Unconstrained | -13219.59 | 105 | BG | 0.00160 (73%) | 0.821 (26%) | 14.9 (0.19%) |  |  | |  |
|  |  |  |  | FG | 0.0549 (92%) | 0.0916 (7.5%) | 287 (0.40%) | 0.0239* | 0.3107 | |  |
|  | Constrained | -13223.33 | 104 | BG | 0.0257 (80%) | 1.00 (20%) | 16.8 (0.13%) |  |  | |  |
|  |  |  |  | FG | 0.0428 (0.0%) | 0.00 (89%) | 1.00 (11%) |  |  | |  |
| **Ancestral Euungulata** | |  |  |  |  |  |  |  |  | |  |
| ***PDE6B*** | Unconstrained | -15970.49 | 95 | BG | 0.0394 (92%) | 0.0614 (5.1%) | 1.94 (2.4%) |  |  | |  |
|  |  |  |  | FG | 0.00 (96%) | 0.00 (4.2%) | 10000 (0.15%) | 0.0216* | 0.2592 | |  |
|  | Constrained | -15974.33 | 94 | BG | 0.0398 (93%) | 0.0609 (5.1%) | 2.01 (2.3%) |  |  | |  |
|  |  |  |  | FG | 0.0347 (0.0%) | 0.00 (92%) | 1.00 (8.1%) |  |  | |  |
| ***RH1*** | Unconstrained | -7036.11 | 103 | BG | 0.0226 (96%) | 0.599 (3.2%) | 16.2 (0.30%) |  |  | |  |
|  |  |  |  | FG | 0.00 (93%) | 0.00 (5.9%) | 726 (0.70%) | 0.0030** | 0.0390* | |  |
|  | Constrained | -7041.92 | 102 | BG | 0.0228 (96%) | 0.592 (3.3%) | 16.6 (0.29%) |  |  | |  |
|  |  |  |  | FG | 0.0129 (0.0%) | 0.00 (90%) | 1.00 (10%) |  |  | |  |
| **Cetartiodactyla** |  |  |  |  |  |  |  |  |  | |  |
| ***CNGB1*** | Unconstrained | -10693.44 | 83 | BG | 0.0281 (86%) | 0.159 (8.9%) | 1.81 (5.4%) |  |  | |  |
|  |  |  |  | FG | 0.144 (90%) | 0.507 (9.8%) | 289 (0.68%) | 0.0023** | 0.0299* | |  |
|  | Constrained | -10699.50 | 82 | BG | 0.0317 (86%) | 0.149 (8.7%) | 1.87 (5.1%) |  |  | |  |
|  |  |  |  | FG | 0.00 (77%) | 0.00 (2.1%) | 1.00 (21%) |  |  | |  |
| ***GRK7*** | Unconstrained | -13215.86 | 105 | BG | 0.0240 (75%) | 0.434 (15%) | 1.58 (9.5%) |  |  | |  |
|  |  |  |  | FG | 0.196 (88%) | 0.755 (11%) | 576 (1.2%) | 0.0001** | 0.0013** | |  |
|  | Constrained | -13224.90 | 104 | BG | 0.0247 (76%) | 0.438 (15%) | 1.58 (9.4%) |  |  | |  |
|  |  |  |  | FG | 0.00 (69%) | 0.931 (0.0%) | 1.00 (31%) |  |  | |  |
| ***SWS1*** | Unconstrained | -8054.16 | 109 | BG | 0.164 (90%) | 0.178 (7.9%) | 3.05 (2.0%) |  |  | |  |
|  |  |  |  | FG | 0.00 (86%) | 0.00 (8.0%) | 23.6 (5.8%) | 0.0189* | 0.2457 | |  |
|  | Constrained | -8058.13 | 108 | BG | 0.167 (90%) | 0.159 (7.8%) | 3.09 (2.0%) |  |  | |  |
|  |  |  |  | FG | 0.00 (43%) | 0.00 (10%) | 1.00 (47%) |  |  | |  |
| **Bovidae** |  |  |  |  |  |  |  |  |  | |  |
| ***PDE6H*** | Unconstrained | -1505.96 | 97 | BG | 0.0457 (91%) | 0.0642 (6.4%) | 2.30 (2.6%) |  |  | |  |
|  |  |  |  | FG | 0.00 (88%) | 0.00 (10%) | 964 (1.4%) | 0.0232* | 0.3016 | |  |
|  | Constrained | -1509.72 | 96 | BG | 0.0479 (92%) | 0.0666 (5.8%) | 2.52 (2.4%) |  |  | |  |
|  |  |  |  | FG | 0.00 (7.8%) | 0.00 (81%) | 1.00 (11%) |  |  | |  |
| **Perissodactyla** |  |  |  |  |  |  |  |  |  | |  |
| ***SWS1*** | Unconstrained | -8053.11 | 109 | BG | 0.171 (94%) | 0.211 (4.4%) | 4.03 (1.3%) |  |  | |  |
|  |  |  |  | FG | 0.00 (82%) | 0.00 (4.9%) | 4.92 (13%) | 0.0310* | 0.4030 | |  |
|  | Constrained | -8056.58 | 108 | BG | 0.157 (95%) | 1.00 (4.4%) | 7.65 (0.40%) |  |  | |  |
|  |  |  |  | FG | 0.00 (63%) | 0.00 (2.4%) | 1.00 (35%) |  |  | |  |
| **Carnivora** |  |  |  |  |  |  |  |  |  | |  |
| ***CNGB3*** | Unconstrained | -10025.07 | 105 | BG | 0.00292 (68%) | 0.725 (0.0%) | 0.718 (32%) |  |  | |  |
|  |  |  |  | FG | 0.00 (93%) | 0.00 (4.8%) | 19.4 (2.7%) | 0.0017** | 0.0221* | |  |
|  | Constrained | -10031.42 | 104 | BG | 0.0148 (70%) | 0.751 (0.0%) | 0.747 (30%) |  |  | |  |
|  |  |  |  | FG | 0.00 (81%) | 0.00 (1.4%) | 1.00 (18%) |  |  | |  |
| ***GUCY2D****^a^* | Unconstrained | -11835.43 | 95 | BG | 0.0311 (85%) | 0.441 (15%) | 10.6 (0.50%) |  |  | |  |
|  |  |  |  | FG | 0.0228 (92%) | 0.00 (6.7%) | 10000 (1.5%) | 0.0065** | 0.0780 | |  |
|  | Constrained | -11840.47 | 94 | BG | 0.0311 (84%) | 0.434 (15%) | 10.5 (0.51%) |  |  | |  |
|  |  |  |  | FG | 0.00 (92%) | 0.00 (1.0%) | 1.00 (7.4%) |  |  |  | |
| **Common ancestors (Carnivora + Pholidota)** | | |  |  |  |  |  |  |  |  | |
| ***PDE6C*** | Unconstrained | -17479.54 | 109 | BG | 0.0153 (78%) | 0.425 (14%) | 1.40 (7.6%) |  |  |  | |
|  |  |  |  | FG | 1.00 (96%) | 1.00 (4.3%) | 3440 (0.19%) | 0.0006** |  | 0.0072** | |
|  | Constrained | -17486.99 | 108 | BG | 0.0180 (79%) | 0.416 (13%) | 1.40 (7.9%) |  |  |  | |
|  |  |  |  | FG | 1.00 (8.1%) | 0.897 (0.0%) | 1.00 (92%) |  |  |  | |

log L,  log-likelihood values, # par., the number of parameters, corrected *P*-value, Bonferroni multiple testing correction, p values are corrected by multiplying them by the number of tested branches of each gene. Significance level is *P* < 0.05. ^a^ shows the sequences of *GUCY2D* gene is unavailable in Feliformia, and only the branch leading to Caniformia was analyzed. **P < 0.05, **P < 0.01*

**Supplementary Table S5 Positively selected genes identified by BS-REL.** Only branches of interest with positively selected genes found are shown.

| **Taxa/gene** | **Mean ω** | **ω1** | **p1** | **ω2** | **p2** | **ω3** | **p3** | **LRT** | ***P*-value** | **Corrected** |
| --- | --- | --- | --- | --- | --- | --- | --- | --- | --- | --- |
|  |  |  |  |  |  |  |  |  |  | ***P*-value** |
| **Carnivora** |  |  |  |  |  |  |  |  |  |  |
| ***CNGB3*** | 0.220156 | 0 | 0.96124 | 0 | 0.013173 | **18.0065** | 0.025587 | 12.0464 | 0.000259** | 0.021017* |
| ***GUCY2D****^a^* | 0.055122 | 0 | 0.957931 | 1 | 0.028275 | **9871.77** | 0.013794 | 10.8204 | 0.000502** | 0.035638* |
| **Ancestral Euungulata** | | |  |  |  |  |  |  |  |  |
| ***RH1*** | 10 | 0 | 0.993427 | 7.50E-08 | 6.95E-05 | **10000** | 0.006504 | 12.1557 | 0.000245** | 0.019086* |
| **Cetartiodactyla** |  |  |  |  |  |  |  |  |  |  |
| ***CNGB1*** | 0.225543 | 0.180221 | 0.960592 | 0.202671 | 0.032764 | **293.679** | 0.006644 | 11.8321 | 0.000291** | 0.017175* |
| ***GRK7*** | 0.402868 | 0.249133 | 0.95954 | 1 | 0.028153 | **582.812** | 0.012307 | 18.4372 | 8.78E-06** | 0.000711** |
| **Common ancestors (Carnivora + Pholidota)** | | |  |  |  |  |  |  |  |  |
| ***PDE6C*** | 1.24697 | 1 | 0.993192 | 1 | 0.004663 | **4270.39** | 0.002145 | 16.3287 | 2.66E-05** | 0.002263** |

p1, p2 and p3 are proportion of sites classified to ω1, ω2 and ω3, LRT: likelihood ratio test statistic, corrected *P* value: after an application of Holm’s multiple testing correction. ^a^ shows the sequences of *GUCY2D* gene is unavailable in Feliformia, and only the branch leading to Caniformia was analyzed. Rectangle shows positive selection signal was lost when phylogenetic uncertainty was taken into account. **P < 0.05, **P < 0.01*

| **Supplementary Table S6 The genes under relatively relaxed selection (*k* < 1) and relatively intensified selection (*k* > 1) in the ancestral bat branch relative to the ancestral Euungulata branch.** The ancestral bat branch was used as test branch and the ancestral Euungulata branch was used as reference branch. The values of the selection intensity parameter (*k*) and its statistical significance were calculated using RELAX.   | **Gene** | **Model** | **log L** | **# par.** | **Branch set** | **ω1** | **ω2** | **ω3** | ***K*** | ***P*-value** | | --- | --- | --- | --- | --- | --- | --- | --- | --- | --- | |  |  |  |  |  |  |  |  |  |  | | ***CNGB3*** |  |  |  |  |  |  |  |  |  | |  | Null | -9939.9 | 184 | Reference branch | 0.129 (4.9%) | 0.130 (94%) | 420 (0.95%) |  |  | |  |  |  |  | Test bra | ch  0.129 (4.9%) | 0.130 (94%) | 420 (0.95%) | 22.21 | 0.0060** | |  | Alternative | -9936.13 | 185 | Reference branch | 0.732 (0.0%) | 0.913 (100%) | 3.38 (0.0%) |  |  | |  |  |  |  | Test branch | 0.000987 (0.0%) | 0.132 (100%) | 5.61E11 (0.0%) |  |  | | ***RCVRN*** |  |  |  |  |  |  |  |  |  | |  | Null | -4575.39 | 192 | Reference branch | 0.655 (97%) | 0.841 (2.8%) | 1.00 (0.0%) |  |  | |  |  |  |  | Test branch | 0.655 (97%) | 0.841 (2.8%) | 1.00 (0.0%) | 39.61 | 0.0000** | |  | Alternative | -4567.1 | 193 | Reference branch | 0.834 (0.0%) | 0.964 (100%) | 1.00 (0.0%) |  |  | |  |  |  |  | Test branch | 0.000757 (0.0%) | 0.234 (100%) | 1.07 (0.0%) |  |  |   ***P < 0.01* | | | | | | | | | | | | | | | | | | |  | |
| --- | --- | --- | --- | --- | --- | --- | --- | --- | --- | --- | --- | --- | --- | --- | --- | --- | --- | --- | --- | --- | --- | --- | --- | --- | --- | --- | --- | --- | --- | --- | --- | --- | --- | --- | --- | --- | --- | --- | --- | --- | --- | --- | --- | --- | --- | --- | --- | --- | --- | --- | --- | --- | --- | --- | --- | --- | --- | --- | --- | --- | --- | --- | --- | --- | --- | --- | --- | --- | --- | --- | --- | --- | --- | --- | --- | --- | --- | --- | --- | --- | --- | --- | --- | --- | --- | --- | --- | --- | --- | --- | --- | --- | --- | --- | --- | --- | --- | --- | --- | --- | --- | --- | --- | --- | --- | --- | --- | --- | --- | --- | --- | --- | --- | --- | --- | --- | --- | --- | --- | --- | --- | --- | --- | --- | --- | --- | --- | --- | --- | --- | --- | --- | --- | --- | --- | --- | --- | --- | --- | --- |
| **Supplementary Table S7 The genes under relatively relaxed selection (*k* < 1) and relatively intensified selection (*k* > 1) in the Carnivora clade**  **relative to the Euungulata clade.** The entire Carnivora clade was used as test clade and the entire Euungulata clade was used as reference clade.  The values of the selection intensity parameter (*k*) and its statistical significance were calculated using RELAX. | | | | | | | | | | | | | | | | | | | | |
| **Gene** | **Model** | **log L** | | | **# par.** | | **Branch set** | | **ω1** | **ω2** | | **ω3** | | ***K*** | | | ***P*-value** | | |  |
|  |  |  | | |  | |  | |  |  | |  | |  | | |  | | |  |
| **Dim-light vision genes** | | | | |  | |  | |  |  | |  | |  | | |  | | |  |
|  |  |  | | |  | |  | |  |  | |  | |  | | |  | | |  |
| ***PDE6G*** |  |  | | |  | |  | |  |  | |  | |  | | |  | | |  |
|  | Null | -1299.83 | | | 124 | | Reference branch | | 0.0160 (88%) | 0.366 (12%) | | 1.00 (0.0%) | | | | |  | | |  |
|  |  |  | | |  | | Test branch | | 0.0160 (88%) | 0.366 (12%) | | 1.00 (0.0%) | | 9.84 | | | 0.0071** | | |  |
|  | Alternative | -1296.20 | | | 125 | | Reference branch | | 0.0335 (86%) | 0.434 (13%) | | 1.10 (0.80%) | | | | |  | | |  |
|  |  |  | | |  | | Test branch | | 3.07E-15 (86%) | 0.000269 (13%) | | 2.65 (0.80%) | | | | |  | | |  |
| ***GNB1*** |  |  | | |  | |  | |  |  | |  | |  | | |  | | |  |
|  | Null | -5266.20 | | | 134 | | Reference branch | | 0.00113 (2.9%) | 0.00305 (97%) | | 3.53 (0.17%) | | | | |  | | |  |
|  |  |  | | |  | | Test branch | | 0.00113 (2.9%) | 0.00305 (97%) | | 3.53 (0.17%) | | 3.74 | | | 0.0374* | | |  |
|  | Alternative | -5264.03 | | | 135 | | Reference branch | | 0.00725 (99%) | 0.0600 (1.4%) | | 4.55 (0.026%) | | | | |  | | |  |
|  |  |  | | |  | | Test branch | | 9.98E-9 (99%) | 0.0000269 (1.4%) | | 288 (0.026%) | | | | |  | | |  |
| ***GRK1*** |  |  | | |  | |  | |  |  | |  | |  | | |  | | |  |
|  | Null | -14324.46 | | | 120 | | Reference branch | | 0.0215 (85%) | 0.623 (15%) | | 1.00 (0.0%) | | | | |  | | |  |
|  |  |  | | |  | | Test branch | | 0.0215 (85%) | 0.623 (15%) | | 1.00 (0.0%) | | 1.76 | | | 2.1236E-5** | | |  |
|  | Alternative | -14315.42 | | | 121 | | Reference branch | | 0.0374 (85%) | 0.687 (15%) | | 1.00 (0.0%) | | | | |  | | |  |
|  |  |  | | |  | | Test branch | | 0.00305 (85%) | 0.516 (15%) | | 1.00 (0.0%) | | | | |  | | |  |
| ***PDE6A*** |  |  | | |  | |  | |  |  | |  | |  | | |  | | |  |
|  | Null | -16088.60 | | | 128 | | Reference branch | | 0.0496 (95%) | 0.577 (5.1%) | | 7.81 (0.24%) | | | | |  | | |  |
|  |  |  | | |  | | Test branch | | 0.0496 (95%) | 0.577 (5.1%) | | 7.81 (0.24%) | | 1.55 | | | 0.0426* | | |  |
|  | Alternative | -16086.50 | | | 129 | | Reference branch | | 0.0128 (87%) | 0.699 (12%) | | 15.3 (0.034%) | | | | |  | | |  |
|  |  |  | | |  | | Test branch | | 0.00115 (87%) | 0.573 (12%) | | 69.3 (0.034%) | | | | |  | | |  |
| ***RH1*** |  |  | | |  | |  | |  |  | |  | |  | | |  | | |  |
|  | Null | -7009.14 | | | 128 | | Reference branch | | 0.0155 (95%) | 0.851 (4.5%) | | 18.1 (0.33%) | | | | |  | | |  |
|  |  |  | | |  | | Test branch | | 0.0155 (95%) | 0.851 (4.5%) | | 18.1 (0.33%) | | 0.00 | | | 0.0028** | | |  |
|  | Alternative | -7004.66 | | | 129 | | Reference branch | | 0.00 (94%) | 0.958 (5.9%) | | 19.8 (0.50%) | | | | |  | | |  |
|  |  |  | | |  | | Test branch | | 0.00 (94%) | 1.00 (5.9%) | | 1.00 (0.50%) | | | | |  | | |  |
|  |  |  | | |  | |  | |  |  | |  | |  | | |  | | |  |
| **Bright-light vision genes** | | | | |  | |  | |  |  | |  | |  | | |  | | |  |
|  |  |  | | |  | |  | |  |  | |  | |  | | |  | | |  |
| ***GUCA1C*** |  |  | | |  | |  | |  |  | |  | |  | | |  | | |  |
|  | Null | -1556.96 | | | 110 | | Reference branch | | 0.00 (77%) | 0.000101 (0.0%) | | 2.72 (23%) | | | | |  | | |  |
|  |  |  | | |  | | Test branch | | 0.00 (77%) | 0.000101 (0.0%) | | 2.72 (23%) | | 0.00 | | | 0.0000** | | |  |
|  | Alternative | -1548.39 | | | 111 | | Reference branch | | 0.00 (76%) | 0.839 (0.0%) | | 4.91 (24%) | | | | |  | | |  |
|  |  |  | | |  | | Test branch | | 0.00 (76%) | 1.00 (24%) | | 1.00 (0.0%) | | | | |  | | |  |
| ***SWS1*** |  |  | | |  | |  | |  |  | |  | |  | | |  | | |  |
|  | Null | -7953.87 | | | 138 | | Reference branch | | 0.00 (86%) | 0.000100 (0.20%) | | 2.16 (14%) | | | | |  | | |  |
|  |  |  | | |  | | Test branch | | 0.00 (86%) | 0.000100 (0.20%) | | 2.16 (14%) | | 0.26 | | | 0.0072* | | |  |
|  | Alternative | -7950.26 | | | 139 | | Reference branch | | 0.0000192 (86%) | 0.000100 (1.4%) | | 2.92 (12%) | | | | |  | | |  |
|  |  |  | | |  | | Test branch | | 0.0614 (86%) | 0.0939 (1.4%) | | 1.32 (12%) | | | | |  | | |  |
| ***CNGB3*** |  |  | | |  | |  | |  |  | |  | |  | | |  | | |  |
|  | Null | -9993.62 | | | 134 | | Reference branch | | 0.00640 (86%) | 0.883 (12%) | | 4.05 (2.1%) | | | | |  | | |  |
|  |  |  | | |  | | Test branch | | 0.00640 (86%) | 0.883 (12%) | | 4.05 (2.1%) | | 3.39 | | | 0.0041** | | |  |
|  | Alternative | -9989.51 | | | 135 | | Reference branch | | 0.00 (88%) | 1.00 (0.63%) | | 1.30 (11%) | | | | |  | | |  |
|  |  |  | | |  | | Test branch | | 0.00 (88%) | 1.00 (0.63%) | | 2.46 (11%) | | | | |  | | |  |
| ***GNAT2*** |  |  | | |  | |  | |  |  | |  | |  | | |  | | |  |
|  | Null | -5847.05 | | | 134 | | Reference branch | | 0.0810 (96%) | 0.0823 (3.5%) | | 31.6 (0.037%) | | | | |  | | |  |
|  |  |  | | |  | | Test branch | | 0.0810 (96%) | 0.0823 (3.5%) | | 31.6 (0.037%) | | 1.33 | | | 0.0342* | | |  |
|  | Alternative | -5844.81 | | | 135 | | Reference branch | | 0.102 (97%) | 0.266 (3.0%) | | 13.9 (0.070%) | | | | |  | | |  |
|  |  |  | | |  | | Test branch | | 0.0485 (97%) | 0.173 (3.0%) | | 32.6 (0.070%) | | | | |  | | |  |
| ***CNGA3*** |  |  | | |  | |  | |  |  | |  | |  | | |  | | |  |
|  | Null | -11447.68 | | | 144 | | Reference branch | | 0.0332 (87%) | 0.609 (13%) | | 1.00 (0.0%) | | | | |  | | |  |
|  |  |  | | |  | | Test branch | | 0.0332 (87%) | 0.609 (13%) | | 1.00 (0.0%) | | 1.42 | | | 0.0053** | | |  |
|  | Alternative | -11443.81 | | | 145 | | Reference branch | | 0.0579 (89%) | 0.677 (9.2%) | | 1.00 (1.5%) | | | | |  | | |  |
|  |  |  | | |  | | Test branch | | 0.0175 (89%) | 0.575 (9.2%) | | 1.00 (1.5%) | | | | |  | | |  |
| ***PDE6C*** |  |  | | |  | |  | |  |  | |  | |  | | |  | | |  |
|  | Null | -17451.30 | | | 140 | | Reference branch | | 0.0207 (90%) | 1.00 (3.1%) | | 1.58 (6.9%) | | | | |  | | |  |
|  |  |  | | |  | | Test branch | | 0.0207 (90%) | 1.00 (3.1%) | | 1.58 (6.9%) | | 1.94 | | | 0.0445* | | |  |
|  | Alternative | -17449.28 | | | 141 | | Reference branch | | 0.0599 (90%) | 0.949 (5.8%) | | 1.37 (4.6%) | | | | |  | | |  |
|  |  |  | | |  | | Test branch | | 0.00426 (90%) | 0.904 (5.8%) | | 1.85 (4.6%) | | | | |  | | |  |
| ***LWS*** |  |  | | |  | |  | |  |  | |  | |  | | |  | | |  |
|  | Null | -4922.60 | | | 82 | | Reference branch | | 0.0155 (87%) | 0.615 (13%) | | 1.00 (0.0%) | | | | |  | | |  |
|  |  |  | | |  | | Test branch | | 0.0155 (87%) | 0.615 (13%) | | 1.00 (0.0%) | | 2.81 | | | 0.0005** | | |  |
|  | Alternative | -4916.54 | | | 83 | | Reference branch | | 0.00 (80%) | 0.589 (20%) | | 1.00 (0.0%) | | | | |  | | |  |
|  |  |  | | |  | | Test branch | | 0.00 (80%) | 0.227 (20%) | | 1.00 (0.0%) | | | | |  | | |  |
|  |  |  | | |  | |  | |  |  | |  | |  | | |  | | |  |
| **Photoresponse recovery genes** | | | | | | |  | |  |  | |  | |  | | |  | | |  |
|  |  |  | | |  | |  | |  |  | |  | |  | | |  | | |  |
| ***RGS9*** |  |  | | |  | |  | |  |  | |  | |  | | |  | | |  |
|  | Null | -8180.33 | | | 124 | | Reference branch | | 0.00 (90%) | 0.951 (10%) | | 1.12 (0.0%) | | | | |  | | |  |
|  |  |  | | |  | | Test branch | | 0.00 (90%) | 0.951 (10%) | | 1.12 (0.0%) | | 23.75 | | | 3.0592E-6** | | |  |
|  | Alternative | -8169.44 | | | 125 | | Reference branch | | 0.00 (86%) | 0.896 (13%) | | 1.06 (1.2%) | | | | |  | | |  |
|  |  |  | | |  | | Test branch | | 0.00 (86%) | 0.0741 (13%) | | 3.71 (1.2%) | | | | |  | | |  |
| ***GUCY2F*** |  |  | | |  | |  | |  |  | |  | |  | | |  | | |  |
|  | Null | -19198.83 | | | 116 | | Reference branch | | 0.175 (88%) | 0.910 (11%) | | 2.40 (1.2%) | | | | |  | | |  |
|  |  |  | | |  | | Test branch | | 0.175 (88%) | 0.910 (11%) | | 2.40 (1.2%) | | 0.58 | | | 0.0009** | | |  |
|  | Alternative | -19193.35 | | | 117 | | Reference branch | | 0.0387 (73%) | 0.555 (14%) | | 1.00 (13%) | | | | |  | | |  |
|  |  |  | | |  | | Test branch | | 0.150 (73%) | 0.710 (14%) | | 1.00 (13%) | | | | |  | | |  |
| **P < 0.05, **P < 0.01* | |  | | |  | |  | |  |  | |  | |  | | |  | | |  |
| **Supplementary Table S8 The genes under relatively relaxed selection (*k* < 1) and relatively intensified selection (*k* > 1) in the Euungulata clade relative to the Carnivora clade.** The entire Euungulata clade was used as test clade and the entire Carnivora clade was used as reference clade. The values of the selection intensity parameter (*k*) and its statistical significance were calculated using RELAX. | | | | | | | | | | | | | | | | | |  | | |
| **Gene** | **Model** | | **log L** | **# par.** | | **Branch set** | | **ω1** | | | **ω2** | | **ω3** | | ***K*** | **P-value** | |  | | |
|  |  | |  |  | |  | |  | | |  | |  | |  |  | |  | | |
| **Dim-light vision genes** | | | |  | |  | |  | | |  | |  | |  |  | |  | | |
|  |  | |  |  | |  | |  | | |  | |  | |  |  | |  | | |
| ***PDE6G*** |  | |  |  | |  | |  | | |  | |  | |  |  | |  | | |
|  | Null | | -1299.80 | 124 | | Reference branch | | 0.00 (77%) | | | 0.253 (23%) | | 1.00 (0.0%) | |  |  | |  | | |
|  |  | |  |  | | Test branch | | 0.00 (77%) | | | 0.253 (23%) | | 1.00 (0.0%) | | 0.31 | 0.0076** | |  | | |
|  | Alternative | | 1296.23 | 125 | | Reference branch | | 0.000000875 (74%) | | | 0.0189 (25%) | | 2.27 (0.62%) | |  |  | |  | | |
|  |  | |  |  | | Test branch | | 0.0141 (74%) | | | 0.297 (25%) | | 1.28 (0.62%) | |  |  | |  | | |
| ***GNB1*** |  | |  |  | |  | |  | | |  | |  | |  |  | |  | | |
|  | Null | | -5269.89 | 134 | | Reference branch | | 0.000100 (2.1%) | | | 0.00289 (98%) | | 3.18 (0.19%) | |  |  | |  | | |
|  |  | |  |  | | Test branch | | 0.000100 (2.1%) | | | 0.00289 (98%) | | 3.18 (0.19%) | | 0.38 | 0.0387* | |  | | |
|  | Alternative | | -5267.75 | 135 | | Reference branch | | 0.00000297 (99%) | | | 0.000100 (1.1%) | | 249 (0.025%) | |  |  | |  | | |
|  |  | |  |  | | Test branch | | 0.00759 (99%) | | | 0.0293 (1.1%) | | 8.29 (0.025%) | |  |  | |  | | |
| ***GRK1*** |  | |  |  | |  | |  | | |  | |  | |  |  | |  | | |
|  | Null | | -14324.46 | 120 | | Reference branch | | 0.0223 (85%) | | | 0.627 (15%) | | 1.00 (0.0%) | |  |  | |  | | |
|  |  | |  |  | | Test branch | | 0.0223 (85%) | | | 0.627 (15%) | | 1.00 (0.0%) | | 0.63 | 0.0000** | |  | | |
|  | Alternative | | -14315.47 | 121 | | Reference branch | | 0.00882 (88%) | | | 0.611 (12%) | | 1.00 (0.0%) | |  |  | |  | | |
|  |  | |  |  | | Test branch | | 0.0514 (88%) | | | 0.734 (12%) | | 1.00 (0.0%) | |  |  | |  | | |
| ***PDE6A*** |  | |  |  | |  | |  | | |  | |  | |  |  | |  | | |
|  | Null | | -16088.57 | 128 | | Reference branch | | 0.0526 (96%) | | | 0.708 (3.8%) | | 8.27 (0.22%) | |  |  | |  | | |
|  |  | |  |  | | Test branch | | 0.0526 (96%) | | | 0.708 (3.8%) | | 8.27 (0.22%) | | 0.65 | 0.0414* | |  | | |
|  | Alternative | | -16086.49 | 129 | | Reference branch | | 0.000914 (87%) | | | 0.534 (13%) | | 67.9 (0.034%) | |  |  | |  | | |
|  |  | |  |  | | Test branch | | 0.0103 (87%) | | | 0.663 (13%) | | 15.8 (0.034%) | |  |  | |  | | |
| ***RH1*** |  | |  |  | |  | |  | | |  | |  | |  |  | |  | | |
|  | Null | | -7009.15 | 128 | | Reference branch | | 0.0176 (96%) | | | 0.902 (4.0%) | | 17.9 (0.34%) | |  |  | |  | | |
|  |  | |  |  | | Test branch | | 0.0176 (96%) | | | 0.902 (4.0%) | | 17.9 (0.34%) | | 7.35 | 0.0030** | |  | | |
|  | Alternative | | -7004.74 | 129 | | Reference branch | | 0.00 (94%) | | | 0.997 (5.8%) | | 1.50 (0.49%) | |  |  | |  | | |
|  |  | |  |  | | Test branch | | 0.00 (94%) | | | 0.976 (5.8%) | | 20.0 (0.49%) | |  |  | |  | | |
|  | | | |  | |  | |  | | |  | |  | |  |  | |  | | |
| **Bright-light vision genes** | | | |  |  | | |
|  |  | |  |  | |  | |  | | |  | |  | |  |  | |  | | |
| ***GUCA1C*** |  | |  |  | |  | |  | | |  | |  | |  |  | |  | | |
|  | Null | | -1557.76 | 110 | | Reference branch | | 0.000100 (73%) | | | 1.00 (10%) | | 3.09 (16%) | |  |  | |  | | |
|  |  | |  |  | | Test branch | | 0.000100 (73%) | | | 1.00 (10%) | | 3.09 (16%) | | 12.99 | 0.0000** | |  | | |
|  | Alternative | | -1549.37 | 111 | | Reference branch | | 0.000100 (78%) | | | 0.944 (0.0%) | | 1.13 (22%) | |  |  | |  | | |
|  |  | |  |  | | Test branch | | E-20 (78%) | | | 0.475 (0.0%) | | 5.17 (22%) | |  |  | |  | | |
| ***SWS1*** |  | |  |  | |  | |  | | |  | |  | |  |  | |  | | |
|  | Null | | -7953.88 | 138 | | Reference branch | | 0.00 (87%) | | | 0.0149 (0.0%) | | 2.18 (13%) | |  |  | |  | | |
|  |  | |  |  | | Test branch | | 0.00 (87%) | | | 0.0149 (0.0%) | | 2.18 (13%) | | 3.36 | 0.0074** | |  | | |
|  | Alternative | | -7950.29 | 139 | | Reference branch | | 0.0568 (87%) | | | 0.0819 (0.85%) | | 1.38 (12%) | |  |  | |  | | |
|  |  | |  |  | | Test branch | | 0.0000645 (87%) | | | 0.000221 (0.85%) | | 2.95 (12%) | |  |  | |  | | |
| ***CNGB3*** |  | |  |  | |  | |  | | |  | |  | |  |  | |  | | |
|  | Null | | -9994.55 | 134 | | Reference branch | | 0.0229 (89%) | | | 1.00 (9.5%) | | 4.21 (2.0%) | |  |  | |  | | |
|  |  | |  |  | | Test branch | | 0.0229 (89%) | | | 1.00 (9.5%) | | 4.21 (2.0%) | | 1.45 | 0.0176* | |  | | |
|  | Alternative | | -9991.74 | 135 | | Reference branch | | 0.174 (4.6%) | | | 0.191 (94%) | | 3.42 (1.4%) | |  |  | |  | | |
|  |  | |  |  | | Test branch | | 0.0791 (4.6%) | | | 0.0905 (94%) | | 5.97 (1.4%) | |  |  | |  | | |
| ***GNAT2*** |  | |  |  | |  | |  | | |  | |  | |  |  | |  | | |
|  | Null | | -5847.06 | 134 | | Reference branch | | 0.0810 (97%) | | | 0.0818 (3.4%) | | 31.6 (0.037%) | |  |  | |  | | |
|  |  | |  |  | | Test branch | | 0.0810 (97%) | | | 0.0818 (3.4%) | | 31.6 (0.037%) | | 0.76 | 0.0340* | |  | | |
|  | Alternative | | -5844.81 | 135 | | Reference branch | | 0.0499 (97%) | | | 0.129 (3.1%) | | 32.5 (0.070%) | |  |  | |  | | |
|  |  | |  |  | | Test branch | | 0.103 (97%) | | | 0.213 (3.1%) | | 13.9 (0.070%) | |  |  | |  | | |
| ***CNGA3*** |  | |  |  | |  | |  | | |  | |  | |  |  | |  | | |
|  | Null | | -11447.71 | 144 | | Reference branch | | 0.0367 (88%) | | | 0.647 (12%) | | 1.00 (0.0%) | |  |  | |  | | |
|  |  | |  |  | | Test branch | | 0.0367 (88%) | | | 0.647 (12%) | | 1.00 (0.0%) | | 0.73 | 0.0052** | |  | | |
|  | Alternative | | -11443.81 | 145 | | Reference branch | | 0.0269 (93%) | | | 0.639 (4.2%) | | 1.00 (3.2%) | |  |  | |  | | |
|  |  | |  |  | | Test branch | | 0.0715 (93%) | | | 0.721 (4.2%) | | 1.00 (3.2%) | |  |  | |  | | |
| ***PDE6C*** |  | |  |  | |  | |  | | |  | |  | |  |  | |  | | |
|  | Null | | -17451.29 | 140 | | Reference branch | | 0.0199 (90%) | | | 1.00 (2.8%) | | 1.55 (7.3%) | |  |  | |  | | |
|  |  | |  |  | | Test branch | | 0.0199 (90%) | | | 1.00 (2.8%) | | 1.55 (7.3%) | | 0.68 | 0.0486* | |  | | |
|  | Alternative | | -17449.35 | 141 | | Reference branch | | 0.0218 (91%) | | | 0.483 (3.2%) | | 1.93 (5.5%) | |  |  | |  | | |
|  |  | |  |  | | Test branch | | 0.0730 (91%) | | | 0.608 (3.2%) | | 1.57 (5.5%) | |  |  | |  | | |
| ***LWS*** |  | |  |  | |  | |  | | |  | |  | |  |  | |  | | |
|  | Null | | -4922.59 | 82 | | Reference branch | | 0.0122 (86%) | | | 0.592 (14%) | | 1.00 (0.0%) | |  |  | |  | | |
|  |  | |  |  | | Test branch | | 0.0122 (86%) | | | 0.592 (14%) | | 1.00 (0.0%) | | 0.37 | 0.0005** | |  | | |
|  | Alternative | | -4916.55 | 83 | | Reference branch | | 7.50E-8 (80%) | | | 0.228 (20%) | | 1.00 (0.0%) | |  |  | |  | | |
|  |  | |  |  | | Test branch | | 0.00227 (80%) | | | 0.578 (20%) | | 1.00 (0.0%) | |  |  | |  | | |
|  |  | |  |  | |  | |  | | |  | |  | |  |  | |  | | |
| **Photoresponse recovery genes** | | | | | |  | |  | | |  | |  | |  |  | |  | | |
|  |  | |  |  | |  | |  | | |  | |  | |  |  | |  | | |
| ***RGS9*** |  | |  |  | |  | |  | | |  | |  | |  |  | |  | | |
|  | Null | | -8180.34 | 124 | | Reference branch | | 0.00 (89%) | | | 0.814 (4.3%) | | 1.00 (6.4%) | |  |  | |  | | |
|  |  | |  |  | | Test branch | | 0.00 (89%) | | | 0.814 (4.3%) | | 1.00 (6.4%) | | 0.36 | 0.0000** | |  | | |
|  | Alternative | | -8171.53 | 125 | | Reference branch | | 0.000100 (1.6%) | | | 0.000699 (93%) | | 1.00 (5.2%) | |  |  | |  | | |
|  |  | |  |  | | Test branch | | 0.0352 (1.6%) | | | 0.0714 (93%) | | 1.00 (5.2%) | |  |  | |  | | |
| ***GUCY2F*** |  | |  |  | |  | |  | | |  | |  | |  |  | |  | | |
|  | Null | | -19198.83 | 116 | | Reference branch | | 0.188 (90%) | | | 0.944 (7.9%) | | 2.28 (1.7%) | |  |  | |  | | |
|  |  | |  |  | | Test branch | | 0.188 (90%) | | | 0.944 (7.9%) | | 2.28 (1.7%) | | 1.94 | 0.0009** | |  | | |
|  | Alternative | | -19193.29 | 117 | | Reference branch | | 0.100 (66%) | | | 0.780 (31%) | | 1.04 (3.1%) | |  |  | |  | | |
|  |  | |  |  | | Test branch | | 0.0115 (66%) | | | 0.617 (31%) | | 1.07 (3.1%) | |  |  | |  | | |

**P < 0.05, **P < 0.01*
